# Cabazitaxel-Loaded Nanoparticles Reduce the Invasiveness in Metastatic Prostate Cancer Cells: Beyond the Classical Taxane Function

**DOI:** 10.3390/pharmaceutics15020662

**Published:** 2023-02-16

**Authors:** Jana B. Lampe, Priyanka P. Desai, Amit K. Tripathi, Nirupama A. Sabnis, Zhe Chen, Amalendu P. Ranjan, Jamboor K. Vishwanatha

**Affiliations:** 1 Department of Microbiology, Immunology, and Genetics, University of North Texas Health Science Center, 3500 Camp Bowie Blvd., Fort Worth, TX 76107, USA; 2 Department of Biophysics, University of Texas Southwestern Medical Center, 6001 Forest Park Road, Dallas, TX 75390, USA

**Keywords:** poly(D, L-lactide-co-glycolide) nanoparticles, bone-targeting, epithelial-to-mesenchymal transition (EMT), cabazitaxel, alendronate, prostate cancer

## Abstract

Bone-metastatic prostate cancer symbolizes the beginning of the later stages of the disease. We designed a cabazitaxel-loaded, poly (lactic-co-glycolic acid) (PLGA) nanoparticle using an emulsion-diffusion-evaporation technique. Bis (sulfosuccinimidyl) suberate (BS3) was non-covalently inserted into the nanoparticle as a linker for the conjugation of a bone-targeting moiety to the outside of the nanoparticle. We hypothesized that the nanoparticles would have the ability to inhibit the epithelial-to-mesenchymal transition (EMT), invasion, and migration in prostate cancer cells. Targeted, cabazitaxel-loaded nanoparticles attenuated the EMT marker, Vimentin, and led to an increased E-cadherin expression. These changes impart epithelial characteristics and inhibit invasive properties in cancer progression. Consequently, progression to distant sites is also mitigated. We observed the reduction of phosphorylated Src at tyrosine 416, along with increased expression of phosphorylated cofilin at serine 3. These changes could affect migration and invasion pathways in cancer cells. Both increased p-120 catenin and inhibition in IL-8 expression were seen in targeted, cabazitaxel-loaded nanoparticles. Overall, our data show that the targeted, cabazitaxel-loaded nanoparticles can act as a promising treatment for metastatic prostate cancer by inhibiting EMT, invasion, and migration, in prostate cancer cells.

## 1. Introduction

The most common cancer in men worldwide, after lung cancer, is prostate cancer (PCa) [1,2]. Global data from 2020 suggest that 1.4 million PCa cases were diagnosed, and approximately 375,000 patients died from the disease [2] When PCa is found and treated in the early stages, there is a 100% cure rate However, once the PCa has metastasized to distant sites, the five-year relative survival rated drops to 30% [3,4]. Moreover, the overall survival of patients with bone metastasis was significantly diminished when compared with those without metastasis to the bone [5]. In 90% of advanced prostate cancer, the bone is the primary site of metastasis [6,7]. In addition to shortening life, bone metastasis destroys the quality of life by inflicting acute pain, pathological fractures, spinal cord compression, bone marrow aplasia, and hypercalcemia. Other possible symptoms that result from hypercalcemia include polyuria, fatigue, and gastrointestinal problems [6].

The overall five-year survival rate for patients with bone metastatic PCa is significantly lower than for patients whose cancer is diagnosed early. Unfortunately, there is no suitable screening test, and many men do not seek regular medical care. Because PCa is asymptomatic until the advanced stages, many men do not learn that they have PCa until it has metastasized to distant sites. Additionally, many PCa patients receive androgen-deprivation therapy as the first-line treatment. Eventually, their tumors become androgen-independent, resulting in metastatic disease. Abiraterone and enzalutamide are two anti-androgen treatments commonly used in the clinic [8].

Recently, there has been a paradigm shift in cancer therapeutics research, emphasizing the prevention of metastasis rather than tumor shrinkage. The Food and Drug Administration (FDA) has recently approved metastasis-free survival (MFS) as a benchmark to indicate treatment efficacy during clinical trials for soft tissue carcinoma and non-metastatic, castration-resistant prostate cancer. This emerging approach focuses on targeting the change in cellular phenotype from epithelial-to-mesenchymal, invasion, migration, and metastasis rather than tumor-cell proliferation. In a recent study known as the CARD trial, #NCT02485691, by Sternberg et al., researchers found that CBZ improved median radiographic progression-free survival and overall survival by a significant margin over abiraterone/enzalutamide-treated patients with metastatic castration-resistant prostate cancer (mCRPC) who had formerly been treated with docetaxel [9,10].

Currently, clinically relevant treatments for bone-metastatic prostate cancer are palliative in nature. Metastasis is one of cancer’s most lethal characteristics, yet no drugs are available to prevent metastasis [11,12]. The first-generation taxanes, paclitaxel and docetaxel, have a strong attraction to the P-glycoprotein (P-gp)efflux pump. Thus, a new taxane was synthesized to overcome this problem. Cabazitaxel, though very similar to the taxane docetaxel, offers structural modifications that make it less prone to resistance. The core structure is the same as docetaxel, but two hydroxyl groups are substituted with two methoxy side chains. These methyl groups disable the adenosine-5′-triphosphate (ATP)-dependent efflux pump [13]. Cabazitaxel (CBZ) is antiproliferative and does shrink tumors; however, it has capabilities outside its classical, well-known taxane functions. CBZ also inhibits genes, proteins, and chemokines known to promote metastatic progression [14]. Unfortunately, the clinical use of CBZ is limited due to high systemic toxicity [15]. Treatments targeting tumors require doses that cause severe side-effects and, in many cases, death. CBZ is a clinically approved chemotherapy treatment effective in earlier stages of prostate cancer. 

A strategy to improve the toxicity profile of CBZ is to load it into a NP. Additionally, targeting bone metastasis can reduce the risk of off-target side effects. Specific targeting of bone tumors can be accomplished by conjugating an anionic compound, alendronate (ALN), to the outside of drug-loaded poly(lactic-co-glycolic acid) (PLGA) nanoparticles (NPs). The function of PLGA is to act as a drug delivery vehicle for the CBZ. PLGA is a hydrolyzable, biodegradable polymer that provides slow release of the drug over time. These characteristics enable improved pharmacokinetics because the drugs are prevented from interacting with the biological fluids in the body, and, in turn, prolong the half-life of their cargo [16]. In addition, chemotherapeutic drugs, such as cabazitaxel, have low solubility rates due to their hydrophobic nature. Nanoparticles provide a more effective means of transport for these drugs. ALN carries a 2^−^charge, which has a high affinity to the Ca2^+^ in bone and can successfully be used to target bone metastasis [17]. As the FDA has approved all three components for clinical use, this newly developed formulation has excellent translational potential as a therapy for PCa patients [16]. 

The primary advantages of multi-functional NPs with drug-loading and targeting include improved drug efficacy and decreased off-target side-effects. Previous research from our lab and in other literature has demonstrated that ALN-coated PLGA NPs effectively target PCa and breast cancer bone-metastasis and other bone-related disorders [17,18,19]. In this study, we hypothesize that targeted CBZ-loaded NPs (T CBZ NPs) attenuate genes, proteins, and interleukins involved in cancer cell proliferation, EMT, invasion, migration, inflammation, and therapy resistance. Our findings suggest that T CBZ NPs have an overall effect on the progression of bone metastatic PCa. Hence, it could be a potential anti-metastatic treatment for PCa. 

## 2. Materials and Methods

### 2.1. Cell Culture

PC3 and C4-2B were obtained from ATCC (Manassas, VA, USA) and were confirmed to be mycoplasma-free through PCR. Cells were grown in RPMI 1640 media (Gibco, Grand Island, NY, USA) containing 10% fetal bovine serum (FBS) (Gibco, Grand Island, NY, USA) and 1% Antibiotic-Antimycotic (ABAM) (Gibco, Grands Island, NY, USA). All cells were incubated at 37 °C, under 5% CO_2_, and with 95% relative humidity.

### 2.2. Nanoparticle Formulation

**Figure 1:**
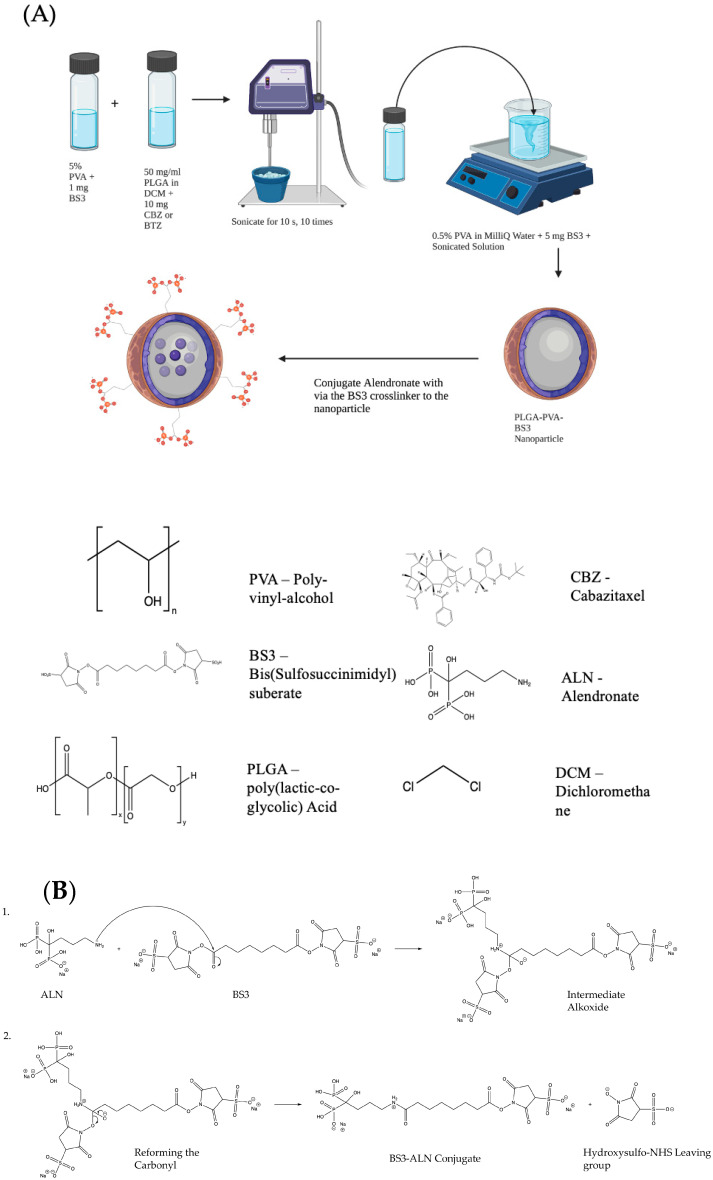
(**A**) Schematic representing the Emulsion-Diffusion-Evaporation Technique for preparing drug-loaded NPs. Schematic created using Biorender.com and ChemDraw. (**B**) Conjugation of Alendronate to the BS3 Crosslinker confers bone-targeting to the drug-loaded nanoparticle. (1) The nitrogen of the 1° amine on the alendronate performs a nucleophilic attack on the carbonyl of the sulfo-NHS ester on the BS3 linker. A proton is taken by a basic species in solution to leave the intermediate alkoxide, which, in (2), reforms the carbonyl, displacing the hydroxysulfo-NHS leaving group to complete the amide formation. Image created with ChemDraw.

An Emulsion-Diffusion-Evaporation Technique was used to prepare the NPs. A schematic of this technique and a model of the final NP are shown in Figure 1A. Briefly, 50 mg of 50:50 PLGA with an inherent viscosity range of 0.55–0.75 dL/g in Hexafluro-2-isopropanol (HFIP) carboxylate End group (nominal) (Durect Corp., Birmingham, AL, USA) was dissolved in 1 mL of MilliQ water and placed on a shaker overnight. Next, 10 mg of CBZ, MedChem Express (Monmouth Junction, NJ, USA), or no drug was added to 1 mL of dichloromethane (DCM) (Sigma-Aldrich, HPLC Plus, St. Louis, MO, USA), and 1 mg of bis(sulfosuccinimidyl) suberate (BS3 linker) (Proteochem, USA Inc., Hurricane, UT, USA) was added to a separate vial with 2 mL of 5% polyvinyl alcohol (PVA) (MW 30,000–70,000; 87–90% hydrolyzed) (Sigma-Aldrich, St. Louis, MO, USA). These two solutions were placed on a rotary stir plate for 15–20 min. Additionally, 5 mg of BS3 linker was added to a 0.5% PVA solution with magnetic stirring for 20 min. The drug/DCM solution and the 5% PVA/BS3 solution were combined and sonicated with an ultrasonic processor UP200H system (Hielscher Ultrasonics GmbH, Teltow, Germany) for ten pulses at 40% amplitude for 10 s on ice. This mixture was then transferred to the 0.5% PVA/BS3 solution and stirred for 4–6 h. The excess solvent was removed by centrifugation and washed twice to remove the excess drug. The NPs were then resuspended in water, followed by lyophilization on an ATR FATR.0 system (USA, Inc., Saint Paul, MN, USA) and stored for further use at −20 °C.

### 2.3. Alendronate Conjugation

Alendronate sodium hydrate (ALN) (Cayman Chemical, Ann Arbor, MI, USA) and lyophilized NPs were prepared with a 1:1 *w*/*w* ratio and suspended separately in 500 μL of phosphate-buffered saline (PBS) (Hyclone, Logan, UT, USA) at room temperature (RT) for 15 min. Conjugation was catalyzed by mixing the two solutions for 1 h at RT. Next, 50 mM tris buffer (pH 7.4) was added for 15 min to stop the reaction. Samples were centrifuged twice at 11,000 rpm for 20 min at RT to remove excess ALN. NPs were resuspended in PBS and stored at 4 °C for further use. The reaction mechanism for the conjugation of ALN to the BS3 linker is shown in Figure 1B. The nitrogen of the 1° amine on the ALN performs a nucleophilic attack on the carbonyl of the Sulfo-NHS ester on the BS3 linker. A proton is taken by a basic species in solution to leave the intermediate alkoxide, which reforms the carbonyl, displacing the hydoxysulfo-NHS leaving group to complete the amide formation.

### 2.4. Nanoparticle Hydrodynamic Size, Polydispersity, and Zeta Potential Measurement

Dynamic light scattering via a Zetasizer Nano ZS instrument (Malvern Panalytical Ltd., Malvern Worcestershire, UK) was used to determine the hydrodynamic particle size, polydispersity index (PDI), and zeta potential (ZP) of targeted NPs and non-targeted NPs both with and without CBZ-loading ± standard deviation (SD). 

### 2.5. Nano-Tracking Analysis

The nanoparticle size characterization was performed by measuring the rate of Brownian motion using a NanoSight NS300 system with NTA version 3.4.4 (Malvern Panalytical, Malvern, UK). Samples were diluted in distilled water and the relative concentration was calculated considering the dilution factor. Samples were analyzed in duplicates using the camera level, slider gain, and slider shutter set as 14, 366, and 1259, respectively. The mode value was considered for sizes of NPs and was measured in nanometer units.

### 2.6. Cryo-Electron Microscopy of Nanoparticles

Three to four microliters of the NP solution were added to Lacey carbon grids (300-mesh; Ted Pella, Inc., Redding, CA, USA) that were negatively glow-discharged for 80 s at 30 mA. Excess sample was removed by blotting once for 4 s with filter paper (Ted Pella, Inc., Redding, CA, USA). Then, the grid was plunge-frozen in liquid ethane cooled by liquid nitrogen using a Vitrobot plunge-freezer (Thermo Fisher Scientific, Waltham, MA, USA). The vitrified samples were imaged using a Glacios 200 kV cryo-transmission electron microscope (cryo-TEM) (Thermo Fisher Scientific) equipped with a Falcon 4 camera (Thermo Fisher Scientific). The Serial EM software v4.0 was used to collect images under low-dose conditions at 92,000× magnification, corresponding to a pixel size of 1 Å/pixel. A total of 80 frames were recorded for each image over an 8 s exposure time.

### 2.7. Determination of Drug Loading and Encapsulation Efficiency

LC/MS was used to determine the drug-loading and encapsulation efficiency of the CBZ NPs. The NPs (500 mg) were weighed out accurately and added to a total of 0.5 mL of solvent (0.5 mL of acetonitrile was added, then 0.2 mL of MilliQ water). Sonication was used to dissolve the lyophilized NPs. A 100 µL solution was pipetted for extraction (n = 4), and two samples were diluted and reinjected to check dilution integrity. The Agilent 6460 QQQ HPLC Mass Spectrometer system (Santa Clara, CA, USA) was equipped with a U.V. detector and reverse-phase C18 column (Allure C-18, 5 μm, 100 mm × 4.6 mm, Restek, Germany) with mobile gradient phase. The diluent was acetonitrile: water (1:1, *v*/*v*), mobile phase A of trifluoracetic acid: water (0.5:1000, *v*/*v*), and mobile phase B of trifluoracetic acid: acetonitrile (0.5:1000, *v*/*v*). Standards and samples were run at a constant flow rate of 1 mL/minute, and the CBZ was quantified by integrating the area under its peak wavelength of 220 nm as compared to the standard curve.

Drug loading (DL) calculations were performed using the equation:

% DL = ((weight of drug in NPs)/(weight of NPs)) × 100%.

Encapsulation Efficiency (EE) was determined using the equation:

% EE = ((actual amount of drug encapsulated in NPs as determined by LC/MS)/(amount of drug used in NP formulation)) × 100%.

### 2.8. Drug Release Kinetics

A dialysis method was used to assess the percentage of total CBZ at various time points and the nanoparticle stability for release in in vivo conditions at physiological pH (pH 7.4). NPs were suspended in MilliQ water at a 1 mg/mL concentration. Exactly 100 μL of CBZ NPs was added to Slide-A-Lyzer Mini Dialysis Units with 3500 kDa MWCO (Life Technologies, Carlsbad, CA, USA) and placed in sink conditions of 14 L of PBS and 0.5% Tween-80 (Sigma Aldrich, St. Louis, MO, USA) at 37 °C with stirring at 50 rpm. Samples were removed at the indicated time points. Acetonitrile was used to recover the NPs and the remaining drug in the dialysis chamber. Liquid Chromatography and Mass Spectrometry (LC/MS) was used to quantify the drug that remained in the NPs by the method mentioned previously. 

### 2.9. Cell Viability

The cytotoxicity of the nanoparticle formulations was assessed by measuring the cell viability of the C4-2B cell line. CBZ dose curves were characterized in C4-2B and PC3 cancer cell lines after 48 and 72 h of treatment. Briefly, cells were seeded in 96-well plates (7500 cells/well—C4-2B and 4000 cells/well—PC3) and incubated in RPMI medium containing 10% FBS overnight at 37 °C in a 5% CO_2_ humidified atmosphere. The cells were treated with a series of concentrations of drugs or NPs, and appropriate controls were used: 4 nM Dimethyl sulfoxide (DMSO) equivalent (MP Biomedicals LLC, Irvine, CA, USA), CBZ 2.5 nM, and T BL NPs 2.5 nM. Cell viability was analyzed via the Abcam Cell Counting Kit 8 (WST-8/CCK-8) (Abcam, Waltham, MA, USA), which measures the activity of mitochondrial dehydrogenases. Optical absorbance was measured at 450 nm on a BioTek Synergy 2 spectrophotometer (Biotek Corp., Milano, Italy) and Gen5 software. Results were expressed as percentage viability ± SD with respect to the control group and performed in triplicate.

### 2.10. Wound-Healing Assay

The effect of the NP treatment on the migration of PC3 cells was assessed with a wound-healing assay. PC3 cells were seeded in 6-well plates and allowed to grow to 80% confluency at 37 °C. A sterile, 200 μL pipette tip was used to create equal-sized wounds in the middle of the culture well. Then, wells were washed once with PBS and were treated with CBZ drug and CBZ-loaded NPs (2.5 nM) in RPMI 1640 media with 10% FBS. The positive control was cells treated with DMSO (MP Biomedicals LLC, Irvine, CA, USA) 4 nM equivalent. Images were taken at 0 h, 18 h, and 24 h. The images were then analyzed with Fiji/ImageJ Software v1.53t. The migration index was calculated using the following formula:

Migration index = [(scratched area)_t=0_ − (scratched area)_t=∆t_/(scratched area)_t=0_] × 100%

To obtain the relative migration displacement, the migration index of the treatment group was normalized to the control group [20].

### 2.11. Transwell Invasion Assay

The invasive propensity of PC3 cells was assessed via a transwell chamber with a polyethylene terephthalate membrane (8 μm pore-sized chambers) (Corning Life Sciences, Durham, NC, USA). Cells were serum-starved for 4 h and pre-treated with DMSO 4 nM equivalent, CBZ 2.5 nM, T BL NP 2.5 nM, NT CBZ NP 2.5 nM, or T CBZ NP 2.5 nM in 0.1% serum for 9 h. Next, 100 μL of medium containing 0.1% serum was added into the upper chamber. Next, 5 × 10^4^ PC3 cells were mixed with 0.1% media, and treatments were added to the upper chamber and only media with 10% serum in the lower chamber. Then, cells were incubated at 37 °C for 24 h and fixed with 4% paraformaldehyde (Affymetrix, Cleveland, OH, USA) for 10 min at RT. Later, cells were washed with PBS and fixed in absolute methanol for 20 min at RT after one PBS wash. Further, cells were stained with 0.25% crystal violet stain for 30 min. The stain was removed, and cells were washed with PBS. Non-migrated cells were scraped off with a cotton swab. Cells were viewed under a microscope and counted by Fiji/Image J software v1.53t by selecting five fields of six images each. The experiment was carried out in triplicates.

### 2.12. RT-q PCR

The changes in the mRNA levels of various genes in PC3 cells upon stimulation with CBZ and its NPs were investigated by RT-qPCR. The EMT genes studied were slug, snail, twist, epithelial cell adhesion molecule (EpCAM), Vimentin, N-cadherin, and E-cadherin. Additionally, genes associated with invasion and migration that were included in this study were Annexin A2 (AnxA2), AKT, migration and invasion enhancer 1 (MIEN1), and the androgen receptor (AR). Briefly, total RNA was purified using the TRIzol reagent (Invitrogen, Waltham, MA, USA) according to the manufacturer’s instructions. Total RNA (2 μg) was reverse-transcribed into cDNA using the Superscript-III First-Strand Synthesis kit (Invitrogen) following the manufacturer’s protocol. RT-qPCR was performed with the SYBR Green Master Mix (BioRad, San Francisco, CA, USA) in a Realplex2 Mastercycler ep gradient S thermal cycler (Eppendorf, Enfield, CT, USA). The PCR program followed for all the genes was: activation at 95 °C for 15 min, followed by 40 cycles of 95 °C for 30 s, 54 °C for 1 min, and 72 °C for 5 s. The reaction was finally put on hold at 4 °C. The primers were synthesized by Integrated DNA Technologies (Coralville, IA, USA). The reference gene for the mRNA levels was 18S. Fold expression was calculated according to the 2^−ΔΔ^ Ct method. Target gene primer sequences are listed in Supplementary Table S2. Images were processed in Fiji/ImageJ software v1.53t and overlaid in Image Lab software v6.1(BioRad, Hercules, CA, USA).

### 2.13. Sodium Dodecyl Sulphate (SDS)-PAGE Western Blot and Immunostaining

Target proteins were analyzed by Western blot. PC3 and C4-2B cells were grown in RPMI media containing 10% FBS in 60 mm plates until they reached 70% confluency. The cells were washed three times with PBS and lysed with Radioimmunoprecipitation Assay (RIPA) lysis buffer (Millipore Sigma, Burlington, MA, USA) (5 M NaCl, 1% Triton X-100, 1 M tris, pH 8.0, SDS, Na-deoxycholate, and distilled water) supplemented with Protease Inhibitor Cocktail Set I (Millipore, Burlington, MA, USA) and Phosphatase Inhibitor Cocktail Set I (Millipore). Cells were kept on ice for 45 min. Subsequently, cells were homogenized and kept at 4 °C with rotary stirring for 2 h. Next, cells were subjected to sonication, ten bursts for 2 s each, followed by centrifugation at 13,000 rpm for 15 min at 4 °C. Protein estimation of the supernatant was performed using the Pierce BCA Protein Assay kit (Thermo Fisher Scientific, Rockford, IL, USA) according to the manufacturer’s suggestions. Equal amounts of proteins were separated on 4–12% Bis-Tris NuPAGE gel (Life Technologies Corporation, Carlsbad, CA, USA) and transferred to the membrane iBlot 2 nitrocellulose membrane mini-Stacks (Invitrogen by Thermo Fisher Scientific). The blots were washed three times with Tris-buffered saline containing 0.1% Tween-20 (TBST) and then incubated on a rocker in 5% bovine serum albumin (BSA) (Sigma, St. Louis, MO, USA) in TBST for 2 h. The blots were washed with TBST and kept with the primary antibodies in 2.5% BSA in TBST overnight on a rocker at 4 °C. Before adding the secondary antibody, the blots were washed three times, incubated for 2 h at RT, washed three times with TBST, and developed using the Immobilon Western Chemiluminescent Horseradish Peroxidase (HRP) substrate (Millipore). Images were visualized using an Innotech Alpha Imager and analyzed on an Alpha View Chem HD2 detection system, AIC software v3.2.2.0 (Cells Biosciences, Inc., Preston, VIC, Australia). Antibodies purchased from Cell Signaling (Danvers, MA, USA) were p-Src (Y416) Cat. #2105S; E-Cadherin (cadherin-1 or CDH1), Cat. #3195T; FAK, Cat. #71433S; p-FAK, Cat. #3284S; (Y925); β-catenin, Cat #8480T; GAPDH, Cat. #5174S; Cofilin, Cat. #5175S; p-cofilin (S3), Cat. #3311; Twist, Cat. #69366S; Vimentin, Cat. #5741T; Slug, Cat. #9585T; Cleaved Caspace-3 (Cl-Csp3), Cat. #9661S; PUMA, Cat. #4973. The antibody c-Src was purchased from Santacruz Biotechnology (Cat. #sc-8056, Dallas, TX, USA), and the antibody, p-120 catenin, was purchased from BD Biosciences (Cat #610133, Franklin Lakes, NJ, USA). Primary antibodies were prepared with a 1:1000 dilution, and secondary antibodies with a 1:2000 dilution. 

### 2.14. IL-8 ELISA Assay

Cells were seeded in a 12-well plate in RPMI media with 10% FBS and 1% ABAM. At confluency, the cells were serum-starved for 6 h and then treated with DMSO 4 nM equivalent, CBZ 2.5 nM, T BL NPs 2.5 nM, NT CBZ NPs 2.5 nM, and T CBZ NPs 2.5 nM in 1% FBS. Aliquots of the supernatant were taken after 6 h. The concentration of IL-8 was assessed using BD OptEIA, Human IL-8 ELISA set, by BD Biosciences (Cat. # 555244, San Diego, CA, USA) according to the manufacturer’s protocol. The absorbance was read at 450 nm after stopping the reaction with 1 M H_3_PO_4_.

### 2.15. Statistical Analysis

Unless otherwise specified, all experiments were performed in triplicate. Statistical analysis was conducted using GraphPad Prism (version 9.4.1, GraphPad Software Inc., USA, La Jolla, CA, USA). Schematic drawings were made using BioRender.com and ChemDraw. Statistical significance was determined by applying either Student’s *t*-test or an ordinary one-way ANOVA followed by Tukey’s multiple comparisons test. Statistical significance was considered as * *p* < 0.05, ** *p* < 0.01, *** *p* < 0.001, **** *p* < 0.0001. The mean ± standard deviation (SD) or standard error of the mean (SEM) was used to express the data. 

## 3. Results

### 3.1. Nanoparticle Characterization

**Figure 2:**
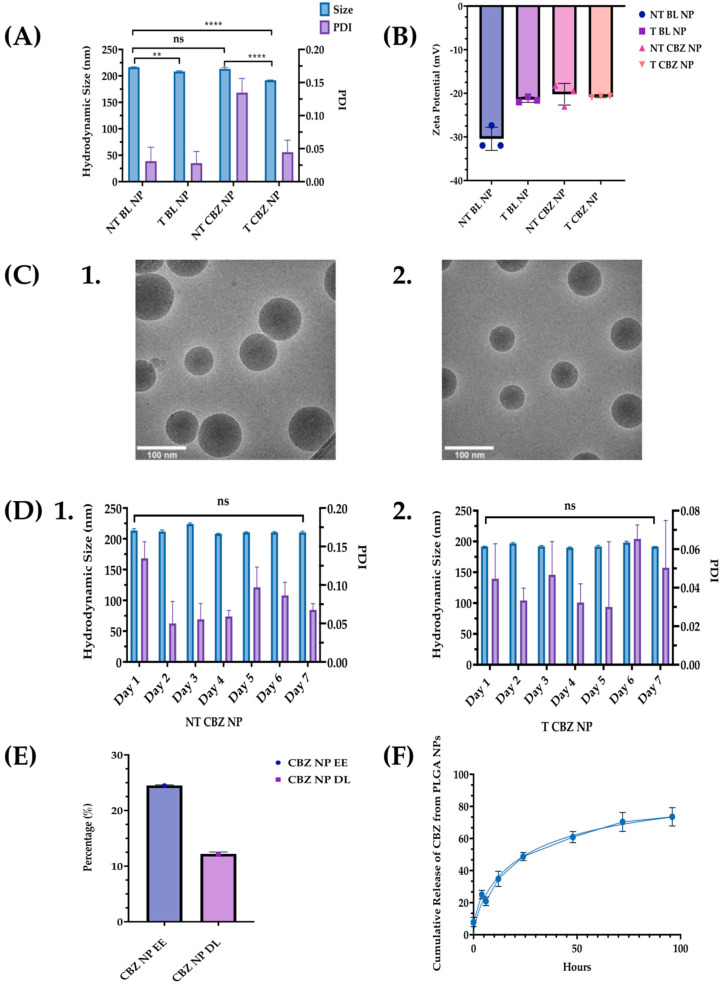
Nanoparticle size, PDI, Zeta Potential, and Cryo-TEM Images. (**A**) A graphical comparison of the size and PDI of non-targeted blank nanoparticles (NT BL NPs), targeted blank nanoparticles (T BL NPs), non-targeted Cabazitaxel-loaded nanoparticles (NT CBZ NPs), and targeted Cabazitaxel-loaded nanoparticles (T CBZ NP); Refer to Table S1 for summary of nanoparticle size, PDI, and ZP; (**B**) Zeta Potentials of the various nanoparticles ± SD; (**C**) Cryo-TEM Images of 1. NT NPs; 2. T NPs. Both nanoparticles are spherical with a relatively smooth surface and a unimodal size distribution. Particle size from cryo-TEM analysis was between 70 and 120 nm. (**D**) Stability of nanoparticles over a seven-day period. 1. NT CBZ NPs; 2. T CBZ NP. (**E**) Encapsulation Efficiency (% EE) was 24.5% and Drug Loading (% DL) of CBZ in PLGA nanoparticles ± SD was 12.195%; (**F**) Cumulative Release of CBZ versus time at pH 7.4 ± SD; A One-Way ANOVA followed by Tukey’s test was used to determine significance. Graphs created with GraphPad Prism 9.4.1. ns = not significant. ** *p* < 0.01, **** *p* < 0.0001 (n = 3).

NPs were characterized for size, ZP, surface morphology, and NP stability. Additionally, DL, EE, and kinetic drug release were determined to evaluate the efficacy of the NP system. NPs were lyophilized using a freezer dryer overnight to avoid degradation and maintain their stability until they were reconstituted in phosphate-buffered saline (PBS) to prepare targeted concentrations for experimental use and characterization (Figure S1). After the NPs were formulated, ALN was conjugated to the outside of the NPs (Figure 1). The hydrodynamic size for non-targeted (without ALN) blank (not drug-loaded) (NT BL NPs) was 216.4 ± 0.833 nm; targeted (with ALN) blank (T BL NPs) was 208.3 ± 1.375 nm; non-targeted CBZ-loaded (NT CBZ NPs) was 213.4 ± 2.884 nm; targeted CBZ-loaded (T CBZ NPs) was 191.8 ± 0.473 nm. The polydispersity index (PDI) of some NPs was less than 0.15, and most were less than 0.05. These PDI values indicate a very homogeneous size population without agglomeration (Figure 2A, Table S1). The ZP of NT BL NPs was −30.43 mV; that of T BL NPs was −21.47 mV; that of NT CBZ NPs was −20.22 mV; that of T CBZ NPs was −20.80 mV (Figure 2B). The cryo-TEM images show that both non-targeted and targeted NPs were spherical with a relatively smooth surface and a unimodal size distribution. The particle size was within the range of 70–120 nm (Figure 2C). Additionally, the hydrodynamic size and PDI were stable for seven days (Figure 2D). The encapsulation efficiency (EE) of CBZ in the NPs was 24.5%, drug loading (DL) was 12.2% (Figure 2E), and CBZ was slowly released over a 96-h period at physiological pH (Figure 2F). Finally, particle size, as determined by Nano Tracking Analysis (NTA), is shown in Figure S2A. The sizes were as follows: NT BL NP, 194.9 nm; T BL NP, 169.7 nm; NT CBZ NP, 168.3 nm; T CBZ NP, 197.9 nm. Figure S2B–E show the representative plots showing the size distribution of nanoparticles using NTA.

### 3.2. Determination of IC50 for Cabazitaxel and Its Targeted and Non-Targeted Nanoparticles in C4-2B PCa Cells

**Figure 3:**
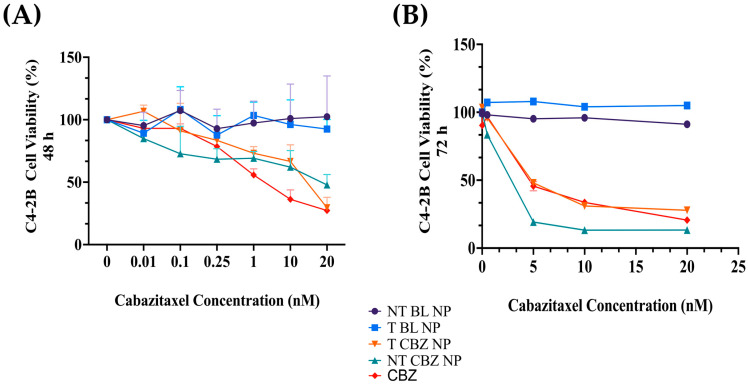
(**A**) Cell viability in C4-2B cell line. The 48 h IC_50_ values in C4-2B cells are as follows: CBZ, 2.8 nM; NT CBZ NP, 24.5 nM; T CBZ NP, 10.52 nM. (**B**) The 72 h IC_50_ values are: CBZ, 4.96; NT CBZ NP, 1.81 nM; T CBZ NP, 5.21 nM (95% confidence intervals (CI)); n = 3. Graphs created with GraphPad Prism 9.4.1. (n = 3).

The toxicity profile of the NPs is a significant criterion in determining their efficacy as a drug delivery system and their potential in clinical, biomedical, and therapeutic applications. We evaluated the free drug and the NPs in C4-2B cells at 48 h and 72 h in a 2D, CCK8 viability assay. The 48 h IC50 values in C4-2B cells were as follows: CBZ, 2.8 nM; NT CBZ NP, 24.5 nM; T CBZ NP, 10.52 nM. The 72 h IC50 values were: CBZ, 4.96; NT CBZ NP, 1.81 nM; T CBZ NP, 5.21 nM (Figure 3). The dose-curves for CBZ alone are shown in Figure S3A for 48 h and Figure S3B for 72 h.

The IC50 values for CBZ in the PC3 cell line were 48 h: 3.8 nM, 72 h: 3.45 nM (Figure S2A). The IC50 values ± SD were calculated using the log (inhibitor) vs. normalized response–variable slope equation in GraphPad Prism 9.4.1. Refer to Figure S2 and Table S3 for CBZ-dose curves in C4-2B and Pc3 PCa cell lines.

### 3.3. Cabazitaxel and Its Targeted Nanoparticles Inhibit Migration and Invasion in PC3 PCa Cells at Sub-IC50 Concentrations

**Figure 4:**
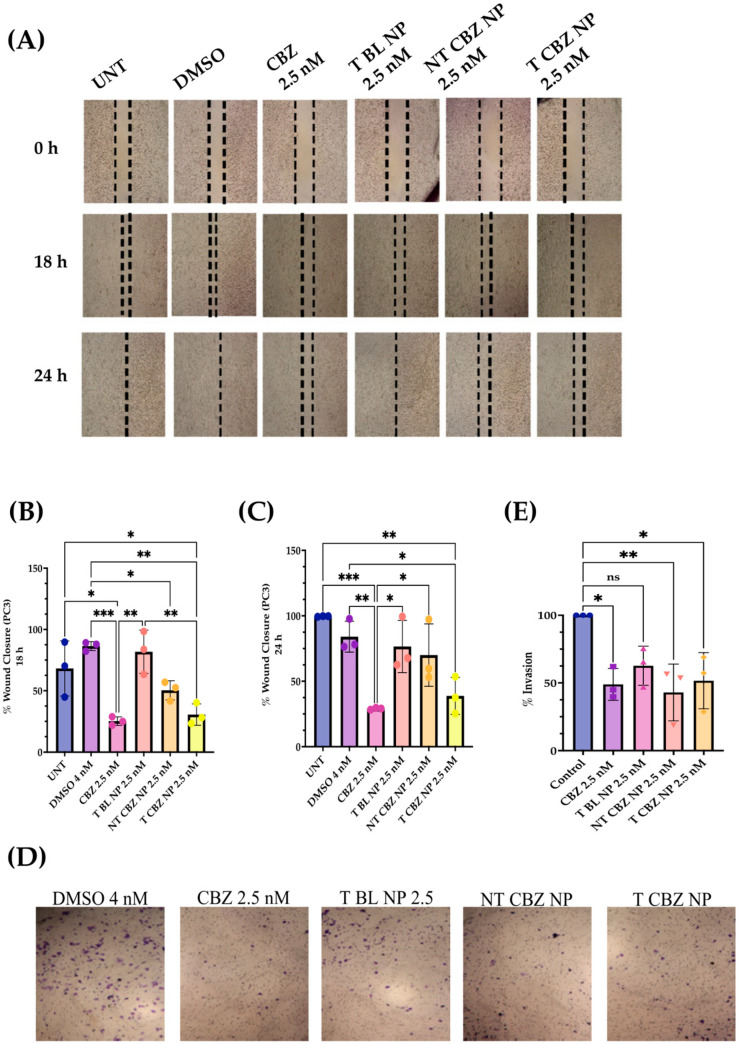
Wound Healing and Transwell Invasion Assays at sub-IC_50_ concentrations. (**A**) Images show inhibition of migration with CBZ-loaded NPs; Graphical representation of wound closure. (**B**) CBZ and CBZ-loaded NPs inhibit migration of PC3 cells after 18 h as compared to untreated; (**C**) 24 h wounds also showed inhibition of migration in CBZ and CBZ-loaded NPs with respect to untreated; (**D**) Transwell Invasion Assay indicated that cell invasion was limited with CBZ and CBZ NP with respect to control after 24 h of treatment; (**E**) graphical representation of % invasion of PC3 cells. Graphs created using GraphPad Prism 9.4.1. ns = not significant, * *p* < 0.05, ** *p* < 0.01, *** *p* < 0.001, (n = 3). Scratch wound and transwell images were created using Fiji/ImageJ software v1.53t.

The CBZ, NT CBZ NPs, and T CBZ NPs significantly inhibited the directional migration and invasion of PC3 cells toward the wound at the sub-IC_50_ concentration of 2.5 nM. The DMSO-treated cells migrated much faster and filled the wound within 24 h compared to the cells treated with CBZ and drug-loaded NPs. Cells at the wound edge of the control groups appeared to have a polarized phenotype, unlike cells treated with drug-loaded NPs. The obvious difference in cell morphology indicated a decrease in motility and, thus, migration (Figure 4A). Scratch wound results are depicted graphically in Figure 4B,C. In addition, the transwell invasion assay showed that at a sub-IC_50_ concentration (2.5 nM), CBZ, NT CBZ NPs, and T CBZ NPs significantly inhibited PC3 cell invasion within 24 h compared to the controls, untreated (UNT), and DMSO 4 nM equivalent (Figure 4D,E).

### 3.4. Cabazitxel and Cabazitaxel-Loaded Nanoparticles Are Involved in the Inhibition of Genes Responsible for Cell Migration and Epithelial–Mesenchymal Transition

**Figure 5:**
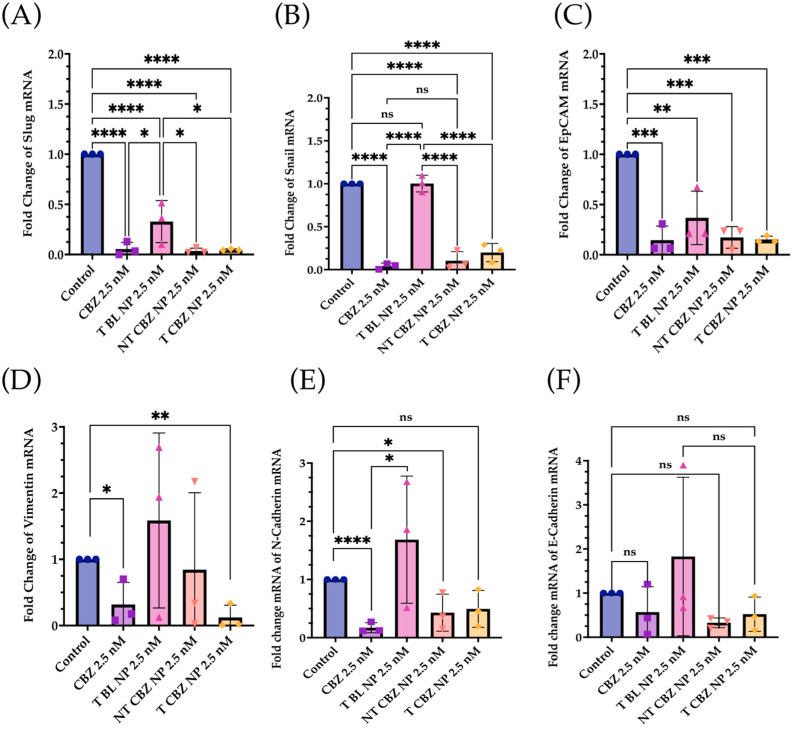
Gene Expression Changes. The mRNA-expression-critical EMT markers and transcription factors were downregulated by CBZ and CBZ NPs at sub-IC_50_ concentrations. (**A**) Slug, (**B**) Snail, (**C**) EpCAM, (**D**) Vimentin, (**E**) N-Cadherin, (**F**) E-Cadherin. E-Cadherin was the only one that did not show significance, but there was a significant increase in its protein expression with T and NT NPs. Graphs created with GraphPad Prism 9.4. ns = not significant, * *p* < 0.05, ** *p* < 0.01, *** *p* < 0.001, **** *p* < 0.0001 (n = 3).

**Figure 6:**
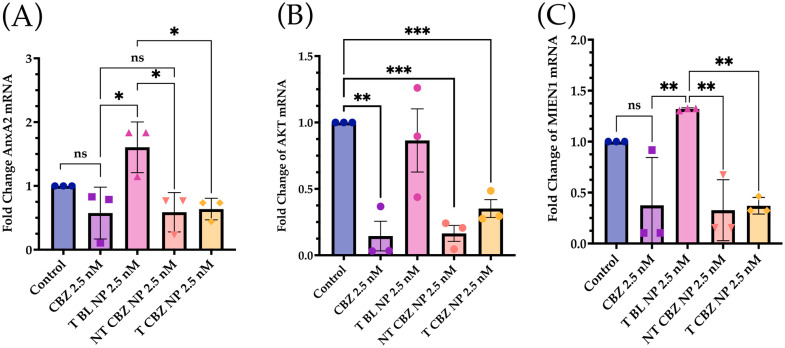
Expression of Genes Associated with Migration and Invasion was attenuated in prostate cancer cells at sub IC_50_ concentrations with respect to control. (**A**) Annexin A2 (AnxA2); (**B**) AKT. (**C**) Migration and Invasion Enhancer 1 (MIEN1) mRNA Expression was downregulated with CBZ and CBZ-loaded NPs at sub IC_50_ concentrations compared to control. Graphs created with GraphPad Prism 9.4.1. ns = not significant, * *p* < 0.05, ** *p* < 0.01, *** *p* < 0.001, (n = 3).

To evaluate the effect of targeted CBZ-loaded NPs on EMT, invasion, and migration in PC3 cells, we tested the expression of genes that mediate cancer metastasis. The cells were treated with the NPs at sub-IC_50_ concentrations to show that T CBZ NPs have an inhibitory effect even at lower concentrations. Interestingly, the CBZ and its NPs inhibited key EMT transcription factors such as slug, snail, vimentin, epithelial cell adhesion molecule (EpCAM), and N-cadherin, which drive the change to metastatic pathogenesis. However, E-cadherin was not significantly inhibited at the gene level (Figure 5A–F). CBZ and CBZ-loaded NPs also downregulated gene-expression-critical proteins involved in migration and invasion. Changes in the mRNA expression of AnxA2, AKT, and MIEN1 are shown in Figure 6A–C. The mRNA status of TWIST1 and the androgen receptor was also tested, but the results were insignificant (Figure S4A,B).

### 3.5. Cabazitaxel and Cabazitaxel-Loaded Nanoparticles Inhibited the Proteins Involved in Migration/Invasion and EMT Signal Transduction Pathways in PC3 Cells

**Figure 7:**
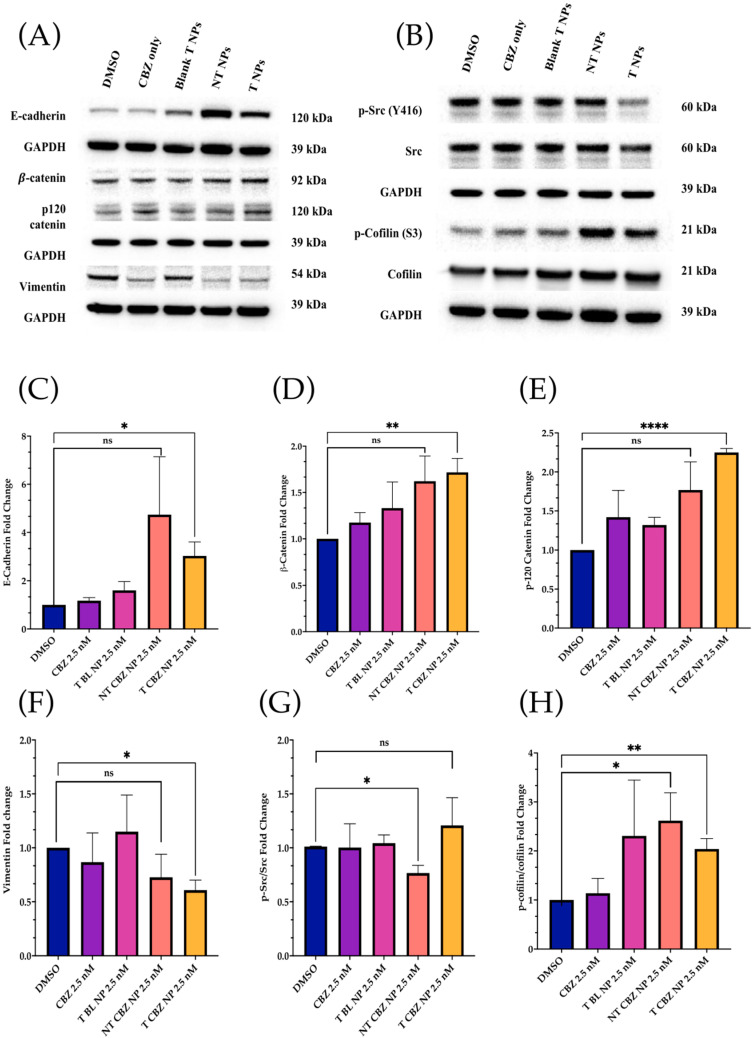
Western Blot Analysis of key signaling proteins. All Western Blot experiments were conducted at sub-IC_50_ concentrations. (**A**) E-cadherin was lower after treatment with CBZ alone but increased with NP treatments. In addition, β-catenin and p-120 catenin expressions were higher with CBZ-loaded NPs, but Vimentin was lower in CBZ and drug-loaded treatments than in control and T BL NPs. (**B**) Activated Src (p-Src) and cofilin (p-cofilin) were lower in drug-alone and drug-loaded NPs with respect to controls. Conversely, total Src and total cofilin were not lower with respect to the controls. (**C**–**H**) Quantification of the Western Blot analysis. All the proteins showed a significant change after T CBZ NPs treatment at 2.5 nM, except that the p-Src/Src ratio only shows significance with NT CBZ NPs. Note that the p-cofilin/cofilin ratio showed significance in both the NT and T CBZ NPs. ns = not significant, * *p* < 0.05, ** *p* < 0.01, **** *p* < 0.0001.

Western blot analysis was performed to check the level of critical proteins involved in migration and invasion. All treatments were at sub-IC_50_ concentrations in PC3 cells. The only exception was the control, DMSO 4 nM equivalent. The results showed that E-cadherin, β-catenin, and p-120 catenin expressions were higher with CBZ-loaded NPs. However, E-cadherin mRNA expression was considerably lower when treated with CBZ alone. Vimentin m RNA expression was also lower when treated with CBZ and CBZ NPs (Figure 7A). Additionally, phosphorylated Rous sarcoma oncogene cellular homolog (p-Src) (Y416) was attenuated with T CBZ NPs. Phospho-cofilin (S3) was higher with NT CBZ NPs and T CBZ NPs with respect to its activated counterpart, cofilin. The loading control, glyceraldehyde-3-phosphate dehydrogenase (GAPDH), was observed to be unchanged (Figure 7B). No significant changes were found in phospho-focal adhesion kinase (p-FAK Y925), FAK, Twist1, and slug at the protein level (Figure S4C,D).

### 3.6. IL-8 Inhibition after Exposure to Cabazitaxel and Its Nanoparticles on PC3 PCa Cells

**Figure 8:**
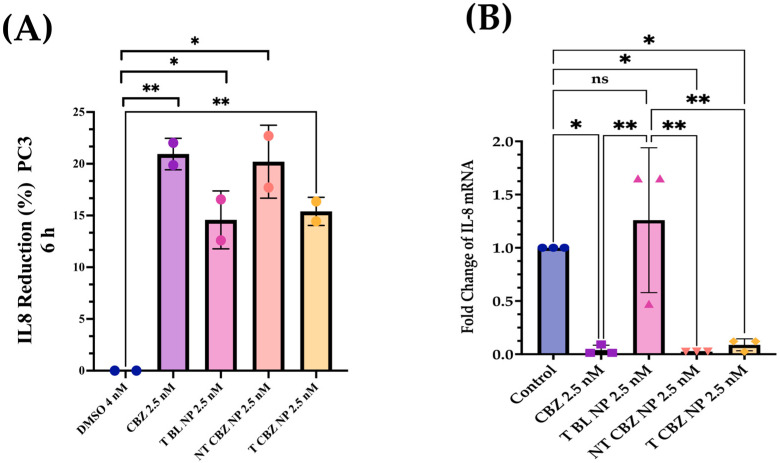
IL-8 ELISA Analysis and Gene Expression Changes. (**A**) An ELISA assay was employed in a PC3 cell line to determine the change in IL-8 secretion with treatment. T BL NPs showed a reduction in the production of IL-8 by 18.9%, NT CBZ NP by 49.8%, and T CBZ by 60.5%. (**B**) IL-8 mRNA expression was lower in CBZ and CBZ-loaded NPs at sub-IC_50_ concentrations compared to control. Graphs are representative of *n* = 3. Graphs created with GraphPad Prism 9.4.1. Statistical significance is considered as ns = not significant, * *p* < 0.05, ** *p* < 0.01.

IL-8 secretion after 6 h of treatment was reduced by 18.9% by T BL NPs, 49.8% by NT CBZ NPs, and 60.5% by T CBZ NPs, with respect to the control in PC3 cells cultured in 1% FBS in RPMI 1640 media (Figure 8A). Furthermore, we found a significant downregulation of the IL-8 mRNA expression in PC3 cells treated with CBZ and CBZ-loaded NPs at sub-IC50 concentrations (Figure 8B).

## 4. Discussion

We report that T CBZ NPs improve the therapeutic efficacy over CBZ in prostate cancer cell lines, to inhibit EMT, invasion, and migration that could lead to further metastasis. The average hydrodynamic size of our T CBZ NPs, 200 nm, is within the range for clinical application, 50 to 500 nm (Figure 2A, Table S1) [21]. The diameter assessed by cryo-TEM is smaller (70–120 nm) than the hydrodynamic size measured by dynamic light scattering (DLS) because the DLS readings consider the ionic interactions of the NPs within the colloidal suspension (Figure 2C). Small NPs (30 nm) are more easily excreted through the kidneys. Larger NPs accumulate in bone and tumor tissues from the enhanced permeation and retention effect (EPR) [22,23,24]. We optimize the size of the NP with its EE and DL, choosing to have a slightly larger size to allow for a higher EE and DL (Figure 2A,E). 

Furthermore, the conjugation of the small molecule, ALN, to the NPs does not affect the NP hydrodynamic size measured by DLS or the size and shape reported by cryo-TEM (Figure 2A,C). ZP is thought to be a predictor of colloidal stability. However, there is some discrepancy in the literature about which ZP values represent stability [25] (Figure 2B). Therefore, we measure the hydrodynamic size daily as a more accurate validation of a consistent size and PDI. The stability of the NPs is also consistent between the targeted and non-targeted NPs. As a result, the CBZ is protected from proteolytic degradation for a prolonged period compared to non-encapsulated CBZ. Additionally, extended drug release has the potential to improve the efficacy of the drug and enables a longer circulation time for the drug to reach its target (Figure 2D). Other advantages of stable NPs are that treatment dose and frequency could be reduced, deleterious effects minimized, and patient compliance to treatment improved [26,27,28].

The EE of the NPs is 24.5%, which is not significantly high. However, this is offset by the exceptionally high DL of 12.2% (Figure 2D). The kinetics drug release study demonstrates that CBZ is discharged in a time-dependent manner from the PLGA NPs (Figure 2E). These data indicate that there will not be a burst of drug exposure that could cause adverse side-effects. Sustained drug release facilitates a steady state of plasma concentrations for a longer time, improves bioavailability, and minimizes quick drug metabolism [29]. 

Our cell viability studies show that cells easily tolerate drug-loaded NPs. The IC_50_ values of the NPs are lower with the NP formulations than with the straight CBZ, indicating that the cells are responsive to a lower concentration of CBZ when introduced by the NPs (Figure 3 and Figure S2, and Table S3). These experiments demonstrate that our BL PLGA NPs are non-toxic to cells. Further experiments were performed with sub-IC_50_ concentrations with respect to these results. 

Two functional assays demonstrate the ability of CBZ, NT CBZ NPs, and T CBZ NPs to inhibit the directional migration and invasion of PC3 cells toward the wound at a concentration of 2.5 nM and above. The untreated and the DMSO-treated cells migrated much faster and fill the wound after 24 h, while the wounds of the CBZ, NT CBZ NP, and T CBZ NP-treated PC3 cells were still visible (Figure 4C). Cell invasion and migration are critical steps in the progression of cancer metastasis [30]. The inhibition of invasion at sub-IC_50_ concentrations indicates that the anti-migratory and anti-invasive abilities of CBZ and CBZ-loaded NPs are not due to the anti-proliferative effect. Instead, the decrease in invasion and migration can be attributed to the effect on critical signaling pathways responsible for cancer progression [31,32]. This inhibition is further confirmed by the light microscopy visualizations of PC3 cells in the wound-healing assay that adhere to the surface and are still adherent after 24 h of treatment. In addition, the images from the transwell invasion assay and its graphical depiction indicate a significant inhibition of invasion by CBZ and its cognate NPs (Figure 4D,E).

The qPCR studies reveal that the CBZ and T CBZ NPs act at the mRNA level on a wide range of migratory and EMT genes (i.e., slug, snail, EpCAM, vimentin, and N-cadherin) (Figure 5A–E) [33]. Despite the significant decrease in the N-cadherin mRNA levels, a hallmark of EMT reversal, there is no substantial change in E-cadherin mRNA expression (Figure 5F). Moreover, the mRNA expression of slug and snail (Figure 5A,B) is reduced by CBZ and its NPs. These transcription factors are known repressors of E-cadherin and promoters of EMT. However, the transcription factor, TWIST1, is decreased by CBZ, and the CBZ-loaded NPs at the mRNA level, but T CBZ NPs do not have a significant effect (Figure S2A). Further studies are needed to investigate the reason for this difference. Additionally, CBZ and NP treatments do not significantly affect androgen receptor (AR) gene expression (Figure S3B).

The mRNA expression of genes associated with invasion and migration is also evaluated. Annexin A2 (AnxA2) has been found to contribute to the homing of PCa cells to the bone marrow and in high levels in high-grade PCa, PC3, and DU145 PCa cell lines [34]. There is also evidence that AnxA2 and its receptor facilitate the growth of tumors in skeletal tissue. As AnxA2 is driven to the bone niche, it alters bone homeostasis, which is prevalent in bone-metastatic disease [34,35]. Our data indicate that NT CBZ NPs and T CBZ NPs can significantly inhibit AnxA2 mRNA expression compared to T BL NPs (Figure 6A), thus providing an added contribution to the anti-invasive and anti-migratory properties of our NP formulation.

Akt, through crosstalk, plays a central role in several critical signaling pathways involved in PCa metastatic progression [36,37,38]. Phospho-Akt at serine 473 reduces the expression of p27Kip1 [39], a cyclin-dependent kinase inhibitor, which leads to prostate cancer tumor progression. The Akt downstream signaling pathway has been implicated in EGFR (Epidermal Growth Factor Receptor) signaling and cell motility in PC3 and DU145 cells for prostate cancer progression [40]. Our qPCR data show a significant attenuation of AKT gene expression in PC3 cells treated with CBZ-loaded NPs with respect to control cells, indicating that CBZ NPs could affect migration or invasion via Akt-mediated pathways.

Besides this, migration and invasion enhancer 1 (MIEN1), responsible for cancer cell migration, is also inhibited (Figure 6C) [40,41]. The inhibition of these genes has a broad and mitigating effect on cancer progression. On the one hand, MIEN1 diminishes the PCa cell migration, and on the other hand, it increases the epithelial cell polarity in the prostate cancer cells [41]. In some gene expression experiments, there is a slight but non-significant inhibition of transcription factors stimulated with blank NPs. The effect of BL NPs can be attributed to the effect of the targeting-moiety, ALN, causing some inhibition in the background [42]. 

As demonstrated by Western blot analysis, changes in protein level also reinforce other data, showing that our CBZ-loaded NPs can inhibit proliferation, EMT, invasion, and migration in a prostate cancer cell line. Loss of E-cadherin is a hallmark of EMT and increases cancer cell motility, and is associated with aggressiveness in many cancers, including prostate cancer [43,44,45]. In response to NT and T CBZ-loaded NPs, the level of E-cadherin increases compared to controls and CBZ alone. This increase indicates that our treatment can restore E-cadherin to levels that stabilize the cell adhesion found in the epithelial phenotype (Figure 7A). To support this conclusion, we also assessed the protein levels of β-catenin and p-120 catenin (p120), which form a complex with E-cadherin. There is a calcium-dependent interaction of the cytoplasmic portion of E-cadherin with p-120 and β-catenin. Attenuation or loss of these components has been shown to play a pivotal role in the progression of EMT and tumorigenesis [45]. These two proteins, β-catenin and p-120 catenin, also increase in response to CBZ and its NPs in our study. 

There is evidence that the elevation of p-120 catenin in the cytoplasm could inhibit nuclear factor κB (NF-κB) activation [46,47]. Lower nuclear factor κB (NF-κB) activation leads to reduced IL-8 mRNA and protein expression, and thus to an anti-inflammatory response [48]. Therefore, we study the IL-8 secretory levels and mRNA expression in NP-treated PC3 cells. Studies have shown that high levels of IL-8 are observed in PCa cells, which is the leading cause of immunotherapy failure. The decrease in the IL-8 levels makes immunotherapy an option in combination with chemotherapy and enhances the positive outcome in PCa patients [47]. In addition, other data have demonstrated a significant increase in serum IL-8 in patients with bone-metastatic disease compared to those with PCa localized in the prostate. Therefore, the reduction in IL-8 secretion and gene expression could be significant. We find that the IL-8 (CXCR8) mRNA transcription is significantly diminished when exposed to CBZ, NT CBZ NPs, and T CBZ NPs in PC3 cells (Figure 8C). There is also a significant decrease in the secretory IL-8 in ELISA experiments (Figure 8A,B). Our study demonstrates that CBZ and CBZ-loaded NPs have an inhibitory effect on the mRNA expression and the secretion of IL-8 at sub-IC50 concentrations. Supplementary Figure S1 shows a potential molecular mechanism based on these findings.

N-cadherin, a mesenchymal marker, functions inversely to E-cadherin, an epithelial marker, in cancer progression. Increased expression of N-cadherin promotes invasive and migratory phenotypes [43,44]. The downregulation of the N-cadherin mRNA expression further supports the hypothesis that the CBZ-loaded NPs have the potential to inhibit EMT (Figure 5E). 

Additionally, the expression of another mesenchymal biomarker, vimentin, is diminished by CBZ and its NPs (Figure 5D). This reduction is significant, given that vimentin has been detected predominantly in poorly differentiated tumors and bone metastases. In other studies, vimentin downregulation substantially attenuated cell motility and invasiveness in a PC3 cell line [48,49]. The effect of the drug-loaded NPs at the mRNA level further substantiates the change in vimentin protein expression (Figure 7A).

We also studied the expression of specific critical proteins involved in cell signaling in prostate cancer progression by our drug-loaded NPs at sub-IC_50_ concentrations. Rous sarcoma oncogene cellular homolog (Src) is a central protein in many signaling pathways involved in PCa. Activated or phosphorylated Src (p-Src) (Y416) has been shown to suppress the association of β-catenin and E-cadherin, leading to increased cell motility. In preclinical studies, Src inhibitors are also known to significantly inhibit cell motility in PC3, DU145, and LNCaP prostate cancer cell lines [50,51]. Additionally, phosphorylation of Src exacerbates the vicious cycle of bone remodeling found in bone tumors and is thought to contribute to bone metastasis. Mouse studies have demonstrated that tumor growth is reduced in metastatic, intratibial tumors through Src inhibition [52]. When PC3 cells are treated with T CBZ NPs, there is a significant decrease in the p-Src/Src ratio (Figure 7B). As it is an essential mediator for oncogenic growth and activation of migration pathways in PC3 cells, we believe that it could be one of the contributing factors in the reduction in cell migration in the wound-healing assay. In its activated form, the protein cofilin induces mobile morphology, cell migration, and cancer metastasis. When cofilin is phosphorylated at serine 3 (p-cofilin) (S3), it becomes inactive [53,54,55,56]. In this study, p-cofilin shows a higher expression with our NPs at sub-IC_50_ concentrations with total cofilin unaffected (Figure 7B). Higher p-cofilin levels demonstrate that NT and T CBZ NPs could also affect actin dynamics, mitigating EMT and cancer metastasis upon treatment. Neither focal adhesion kinase (FAK) nor its activated form (phosphorylated at tyrosine 925 (p-FAK Y925)) are affected by CBZ-loaded NPs (Figure S3C).

Additionally, our CBZ-loaded NT and T NPs have shown effective apoptotic activity in PC3 prostate cancer cells via increased expression of PUMA and by activating and cleaving Caspase-3 (Supplementary Figure S4). Therefore, Supplementary Figure S1 shows the potential molecular mechanism based on these findings. We therefore conclude that CBZ NPs inhibit EMT, invasion, and migration while downregulating signal transduction pathways and transcription factors in metastatic PCa cells [50,51,52,53,54,55,56,57].

NPs have many potential benefits over traditional treatments for advanced prostate cancer. Currently, there are NP therapeutics for other cancers; however, only one NP treatment is clinically available in the United States for prostate cancer. Eligard Neulasta, approved by the FDA in 2002, is a PLGA NP loaded with leuprolide acetate, which lowers testosterone levels. It is a palliative treatment for patients with advanced prostate cancer [58,59,60]. 

As part of our future directions, we would like to characterize the NPs further using NMR, Fourier-transform infrared spectroscopy (FTIR), and X-ray diffraction analysis. Additionally, kinetics release studies could be shown at pH 7.4 and pH 5.5 to show the effect of pH on the release of CBZ from the NPs. The analysis of pro-apoptotic protein markers could also be refined through flow cytometry experiments [61,62,63]. Our novel formulation will need to undergo preclinical testing to verify its in vivo efficacy. For our future in vivo studies, we would like to include in vivo efficacy studies, as well as qPCR and Western blot studies on the serum of or treated mice with respect to the controls. In these studies, we plan to check the same genes and proteins that we studied in vitro.

## 5. Conclusions

In the future, the combination of the functional assays, the gene expression data, and the changes in protein levels show the potential of our T CBZ NP treatment to inhibit the hallmarks of cancer such as EMT, invasion, migration, and inflammation in an animal model. Although more studies are required, our NPs could lead to an anti-proliferative, anti-tumoral, and anti-metastatic treatment with fewer off-target side effects than CBZ alone. In time, these drug-loaded NPs could eventually help fill the gap in the treatment regimen for patients with bone-metastatic prostate cancer. 

## Data Availability

The data will be available from the corresponding author upon reasonable request.

## Supplementary Materials

The following supporting information can be downloaded at: https://www.mdpi.com/article/10.3390/pharmaceutics15020662/s1, Figure S1: Proposed Mechanism p-Src, p-120, and NF-κB Signaling; Figure S2: Nanoparticle Size Characterization by Nano Tracking Analysis (NTA); Figure S3: Cabazitaxel Dose Curves for 48 h and 72 h treatments; Figure S4: Gene and protein expression in PC3 cell line; Figure S5: Western blot showing high expression of Cleaved Caspase-3 (Cl-Csp3) and PUMA in PC3 prostate cancer cells treated with cabazitaxel-loaded non-targeted (NT) and targeted (T) nanoparticles; Table S1: Mean particle size, polydispersity index (PDI), and zeta potential of the various nanoparticles; Table S2: Gene primers for qPCR; Table S3: Summary of IC50 concentrations of Cabazitaxel in C4-2B and PC3 prostate cancer cell lines.

## References

[1] Sun R., Lyu M., Liang S., Ge W., Wang Y., Ding X., Zhang C., Zhou Y., Chen S., Chen L. (2022). A prostate cancer tissue specific spectral library for targeted proteomic analysis. Proteomics.

[2] Withrow D., Pilleron S., Nikita N., Ferlay J., Sharma S., Nicholson B., Rebbeck T.R., Lu-Yao G. (2022). Current and projected number of years of life lost due to prostate cancer: A global study. Prostate.

[3] Xia C., Yu X.Q., Chen W. (2023). Measuring population-level cure patterns for cancer patients in the United States. Int. J. Cancer.

[4] Sayegh N., Swami U., Agarwal N. (2022). Recent Advances in the Management of Metastatic Prostate Cancer. JCO Oncol. Pract..

[5] Ryan C., Stoltzfus K.C., Horn S., Chen H., Louie A.V., Lehrer E.J., Trifiletti D.M., Fox E.J., Abraham J.A., Zaorsky N.G. (2022). Epidemiology of bone metastases. Bone.

[6] Maurizi A., Rucci N. (2018). The Osteoclast in Bone Metastasis: Player and Target. Cancers.

[7] Hu K., Hu X., Duan Y., Li W., Qian J., Chen J. (2022). A Novel Overall Survival Prediction Signature Based on Comprehensive Research in Prostate Cancer Bone Metastases. Front. Med..

[8] Hongo H., Kosaka T., Oya M. (2018). Analysis of cabazitaxel-resistant mechanism in human castration-resistant prostate cancer. Cancer Sci..

[9] Sternberg C.N., Castellano D., de Bono J., Fizazi K., Tombal B., Wülfing C., Kramer G., Eymard J.C., Bamias A., Carles J. (2021). Efficacy and Safety of Cabazitaxel Versus Abiraterone or Enzalutamide in Older Patients with Metastatic Castration-resistant Prostate Cancer in the CARD Study. Eur. Urol..

[10] de Wit R., de Bono J., Sternberg C.N., Fizazi K., Tombal B., Wülfing C., Kramer G., Eymard J.C., Bamias A., Carles J. (2019). Cabazitaxel versus Abiraterone or Enzalutamide in Metastatic Prostate Cancer. N. Engl. J. Med..

[11] Solomon J., Raškova M., Rösel D., Brábek J., Gil-Henn H. (2021). Are We Ready for Migrastatics?. Cells.

[12] Beaver J.A., Kluetz P.G., Pazdur R. (2018). Metastasis-free Survival—A New End Point in Prostate Cancer Trials. N. Engl. J. Med..

[13] Nightingale G., Ryu J. (2012). Cabazitaxel (jevtana): A novel agent for metastatic castration-resistant prostate cancer. Pharm. Ther..

[14] Eryilmaz E.I., Eskiler G.G., Egeli U., Cecener G. (2021). The regulatory effect of cabazitaxel on epithelia-mesenchymal transition in metastatic prostate cancer. J. Can. Res. Ther..

[15] Wan Z., Xie F., Wang L., Zhang G., Zhang H. (2020). Preparation and Evaluation of Cabazitaxel-Loaded Bovine Serum Albumin Nanoparticles for Prostate Cancer. Int. J. Nanomed..

[16] Kucuksayan E., Bozkurt F., Yilmaz M.T., Sircan-Kucuksayan A., Hanikoglu A., Ozben T. (2021). A new combination strategy to enhance apoptosis in cancer cells by using nanoparticles as biocompatible drug delivery carriers. Sci. Rep..

[17] Gdowski A.S., Ranjan A., Sarker M.R., Vishwanatha J.K. (2017). Bone-targeted cabazitaxel nanoparticles for metastatic prostate cancer skeletal lesions and pain. Nanomedicine.

[18] Chen Y., Pan Y., Hu D., Peng J., Hao Y., Pan M., Yuan L., Yu Y., Qian Z. (2021). Recent progress in nanoformulations of cabazitaxel. Biomed. Mater..

[19] Thamake S.I., Raut S.L., Gryczynski Z., Ranjan A.P., Vishwanatha J.K. (2012). Alendronate coated poly-lactic-co-glycolic acid (PLGA) nanoparticles for active targeting of metastatic breast cancer. Biomaterials.

[20] Suarez-Arnedo A., Torres Figueroa F., Clavijo C., Arbeláez P., Cruz J.C., Muñoz-Camargo C. (2020). An image J plugin for the high throughput image analysis of in vitro scratch wound healing assays. PLoS ONE.

[21] Rizvi S.A.A., Saleh A.M. (2018). Applications of nanoparticle systems in drug delivery technology. Saudi Pharm J..

[22] Moghimi S.M., Hunter A.C., Murray J.C. (2001). Long-circulating and target-specific nanoparticles: Theory to practice. Pharm. Rev..

[23] Nakamura Y., Mochida A., Choyke P.L., Kobayashi H. (2016). Nanodrug Delivery: Is the Enhanced Permeability and Retention Effect Sufficient for Curing Cancer?. Bioconjug Chem..

[24] Wu J. (2021). The Enhanced Permeability and Retention (EPR) Effect: The Significance of the Concept and Methods to Enhance Its Application. J. Pers Med..

[25] Bhattacharjee S. (2016). DLS and zeta potential—What they are and what they are not?. J. Control. Release.

[26] Güncüm E., Işıklan N., Anlaş C., Ünal N., Bulut E., Bakırel T. (2018). Development and characterization of polymeric-based nanoparticles for sustained release of amoxicillin—An antimicrobial drug. Artif. Cells Nanomed. Biotechnol..

[27] Jahan S.T., Sadat S.M.A., Walliser M., Haddadi A. (2017). Targeted Therapeutic Nanoparticles: An Immense Promise to Fight against Cancer. J. Drug Deliv..

[28] Pudlarz A., Szemraj J. (2018). Nanoparticles as carriers of proteins, peptides and other therapeutic molecules. Open Life Sci..

[29] Alasvand N., Urbanska A.M., Rahmati M., Saeidifar M., Gungor-Ozkerim P.S., Sefat F., Rajadas J., Mozafari M., Grumezescu A.M. (2017). Chapter 13—Therapeutic nanoparticles for targeted delivery of anticancer drugs. Multifunctional Systems for Combined Delivery, Biosensing and Diagnostics.

[30] van Zijl F., Krupitza G., Mikulits W. (2011). Initial steps of metastasis: Cell invasion and endothelial transmigration. Mutat Res..

[31] Sever R., Brugge J.S. (2015). Signal transduction in cancer. Cold Spring Harb Perspect Med..

[32] Odero-Marah V., Hawsawi O., Henderson V., Sweeney J. (2018). Epithelial-Mesenchymal Transition (EMT) and Prostate Cancer. Adv Exp Med Biol..

[33] Zhu M.L., Horbinski C.M., Garzotto M., Qian D.Z., Beer T.M., Kyprianou N. (2010). Tubulin-targeting chemotherapy impairs androgen receptor activity in prostate cancer. Cancer Res..

[34] Anselmino N., Bizzotto J., Sanchis P., Lage-Vickers S., Ortiz E., Valacco P., Paez A., Labanca E., Meiss R., Navone N. (2020). HO-1 Interactors Involved in the Colonization of the Bone Niche: Role of ANXA2 in Prostate Cancer Progression. Biomolecules.

[35] Lokman N.A., Ween M.P., Oehler M.K., Ricciardelli C. (2011). The role of annexin A2 in tumorigenesis and cancer progression. Cancer Microenviron.

[36] da Silva H.B., Amaral E.P., Nolasco E.L., de Victo N.C., Atique R., Jank C.C., Anschau V., Zerbini L.F., Correa R.G. (2013). Dissecting Major Signaling Pathways throughout the Development of Prostate Cancer. Prostate Cancer..

[37] Kreisberg J.I., Malik S.N., Prihoda T.J., Bedolla R.G., Troyer D.A., Kreisberg S., Ghosh P.M. (2004). Phosphorylation of Akt (Ser473) is an excellent predictor of poor clinical outcome in prostate cancer. Cancer Res..

[38] Ramalingam S., Ramamurthy V.P., Njar V.C.O. (2017). Dissecting major signaling pathways in prostate cancer development and progression: Mechanisms and novel therapeutic targets. J. Steroid Biochem. Mol. Biol..

[39] Walker L., Millena A.C., Strong N., Khan S.A. (2013). Expression of TGFβ3 and its effects on migratory and invasive behavior of prostate cancer cells: Involvement of PI3-kinase/AKT signaling pathway. Clin. Exp. Metastasis.

[40] Kpetemey M., Chaudhary P., Van Treuren T., Vishwanatha J.K. (2016). MIEN1 drives breast tumor cell migration by regulating cytoskeletal-focal adhesion dynamics. Oncotarget.

[41] Kushwaha P.P., Gupta S., Singh A.K., Kumar S. (2019). Emerging Role of Migration and Invasion Enhancer 1 (MIEN1) in Cancer Progression and Metastasis. Front Oncol..

[42] Tuomela J.M., Valta M.P., Väänänen K., Härkönen P.L. (2008). Alendronate decreases orthotopic PC-3 prostate tumor growth and metastasis to prostate-draining lymph nodes in nude mice. BMC Cancer.

[43] Loh C.Y., Chai J.Y., Tang T.F., Wong W.F., Sethi G., Shanmugam M.K., Chong P.P., Looi C.Y. (2019). The E-Cadherin and N-Cadherin Switch in Epithelial-to-Mesenchymal Transition: Signaling, Therapeutic Implications, and Challenges. Cells.

[44] Mendonsa A.M., Bandyopadhyay C., Gumbiner B.M. (2020). p120-catenin phosphorylation status alters E-cadherin mediated cell adhesion and ability of tumor cells to metastasize. PLoS ONE.

[45] Nakajima S., Doi R., Toyoda E., Tsuji S., Wada M., Koizumi M., Tulachan S.S., Ito D., Kami K., Mori T. (2004). N-cadherin expression and epithelial-mesenchymal transition in pancreatic carcinoma. Clin Cancer Res..

[46] Maynard J.P., Ertunc O., Kulac I., Baena-Del Valle J.A., De Marzo A.M., Sfanos K.S. (2020). IL8 Expression Is Associated with Prostate Cancer Aggressiveness and Androgen Receptor Loss in Primary and Metastatic Prostate Cancer. Mol. Cancer Res..

[47] Madan R.A., Palena C. (2021). Behind the IL-8 ball in prostate cancer. Nat. Cancer.

[48] Wang M., Li N., Li J., Ma Y., Li D., Qin L., Wang X., Wu R. (2010). Involvement of p120 in LPS-induced NF-kappaB activation and IL-8 production in human bronchial epithelial cells. Toxicol. Lett..

[49] Satelli A., Li S. (2011). Vimentin in cancer and its potential as a molecular target for cancer therapy. Cell. Mol. Life Sci..

[50] Dave J.M., Bayless K.J. (2014). Vimentin as an integral regulator of cell adhesion and endothelial sprouting. Microcirculation.

[51] Varkaris A., Katsiampoura A.D., Araujo J.C., Gallick G.E., Corn P.G. (2014). Src signaling pathways in prostate cancer. Cancer Metastasis Rev..

[52] Fizazi K. (2007). The role of Src in prostate cancer. Ann. Oncol..

[53] Martellucci S., Clementi L., Sabetta S., Mattei V., Botta L., Angelucci A. (2020). Src Family Kinases as Therapeutic Targets in Advanced Solid Tumors: What We Have Learned so Far. Cancers.

[54] Xu J., Huang Y., Zhao J., Wu L., Qi Q., Liu Y., Li G., Li J., Liu H., Wu H. (2021). Cofilin: A Promising Protein Implicated in Cancer Metastasis and Apoptosis. Front. Cell Dev. Biol..

[55] Bernstein B.W., Bamburg J.R. (2010). ADF/cofilin: A functional node in cell biology. Trends Cell Biol..

[56] Chan C., Beltzner C.C., Pollard T.D. (2009). Cofilin dissociates Arp2/3 complex and branches from actin filaments. Curr. Biol..

[57] Qin L., Qin S., Zhang Y., Zhang C., Ma H., Li N., Liu L., Wang X., Wu R. (2014). p120 modulates LPS-induced NF-κB activation partially through RhoA in bronchial epithelial cells. Biomed. Res. Int..

[58] Wilson A.C., Meethal S.V., Bowen R.L., Atwood C.S. (2007). Leuprolide acetate: A drug of diverse clinical applications. Expert Opin. Investig. Drugs.

[59] Rosario D.J., Davey P., Green J., Greene D., Turner B., Payne H., Kirby M. (2016). The role of gonadotrophin-releasing hormone antagonists in the treatment of patients with advanced hormone-dependent prostate cancer in the UK. World J. Urol..

[60] Wang S., Cheng K., Chen K., Xu C., Ma P., Dang G., Yang Y., Lei Q., Huang H., Yu Y. (2022). Nanoparticle-based medicines in clinical cancer therapy. Nano Today.

[61] Shah K.A., Li G., Song L., Gao B., Huang L., Luan D., Iqbal H., Cao Q., Menaa F., Lee B.-J. (2022). Rizatriptan-Loaded Oral Fast Dissolving Films: Design and Characterizations. Pharmaceutics.

[62] Iqbal H., Razzaq A., Khan N.U., Rehman S.U., Webster T.J., Xiao R., Menaa F. (2022). pH-responsive albumin-coated biopolymeric nanoparticles with lapatinab for targeted breast cancer therapy. Biomater. Adv..

[63] Menaa F. (2013). When Pharma Meets Nano or The Emerging Era of Nano-Pharmaceuticals. Pharmaceut. Anal. Acta.

