# Supplementary material for: Cabazitaxel-Loaded Nanoparticles Reduce the Invasiveness in Metastatic Prostate Cancer Cells: Beyond the Classical Taxane Function
Source: Pharmaceutics. 2023 Feb 16;15(2):662. doi: 10.3390/pharmaceutics15020662 (PMC9967362; doi:10.3390/pharmaceutics15020662)
Supplement: Supplementary file 1 [file pharmaceutics-15-00662-s001.zip › pharmaceutics-2137762-supplementary.pdf]

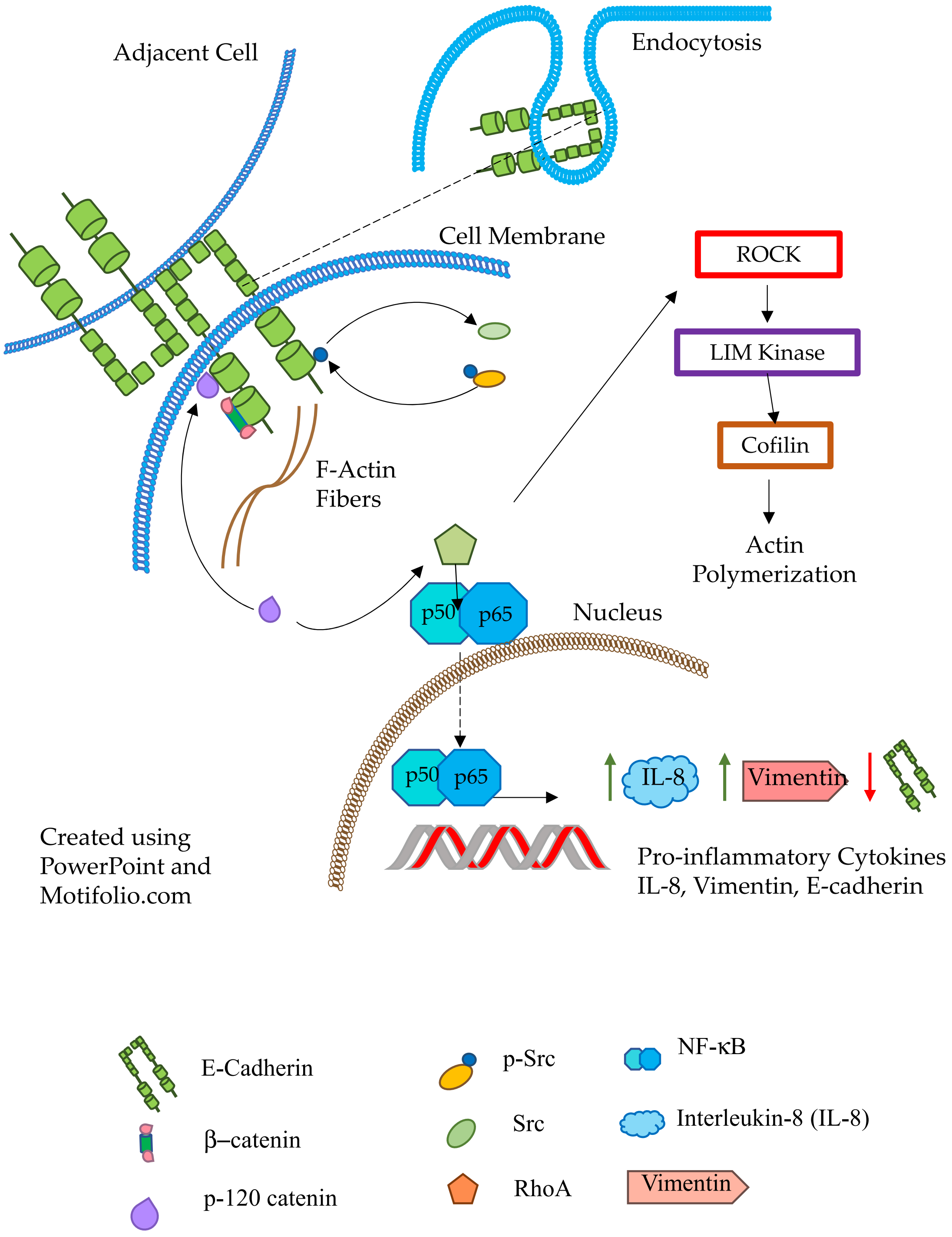

Figure S1. Proposed Mechanism p-Src, p-120, and NF-κB Signaling. (1) The E-cadherin complex comprises E-cadherin, β–catenin, and p-120 catenin. The p-120 catenin stabilized E-cadherin with the cell membrane. (2) p-Src (Y416) phosphorylates E-cadherin at a dileucine motif, causing a conformation change and dismantling of the E-cadherin complex. (3) E-cadherin goes through endocytosis and is marked for either recycling or degradation in lysosomes. (4). Cytosolic p-120 activates RhoA signaling, which, in turn, activates NF-κ B. (5) NF-κ B translocations to the nucleus and (6) elicits transcription of pro-inflammatory cytokines such as IL-8. (7) RhoA can also trigger the ROCK/LIM kinase/cofilin (S3) signaling, which (8) induces changes in actin polymerization that are favorable for motility and migration.

Table S1. Mean particle size, polydispersity index (PDI), and zeta potential of the various nanoparticles.

Groups	Size (nm) ± SD	PDI ± SD	Zeta Potentials (mV) ± SD
**NT BL NP**	216.4 ± 0.83	0.031 ± 0.02	-30.4 ± 2.66
**T BL NP**	208.3 ± 1.37	0.028 ± 0.02	-21.5 ± 0.61
**NT CBZ NP**	213.4 ± 2.88	0.14 ± 0.02	-20.2 ± 2.49
**T CBZ NP**	191.8 ± 0.47	0.05 ± 0.02	-20.8 ± 0.17

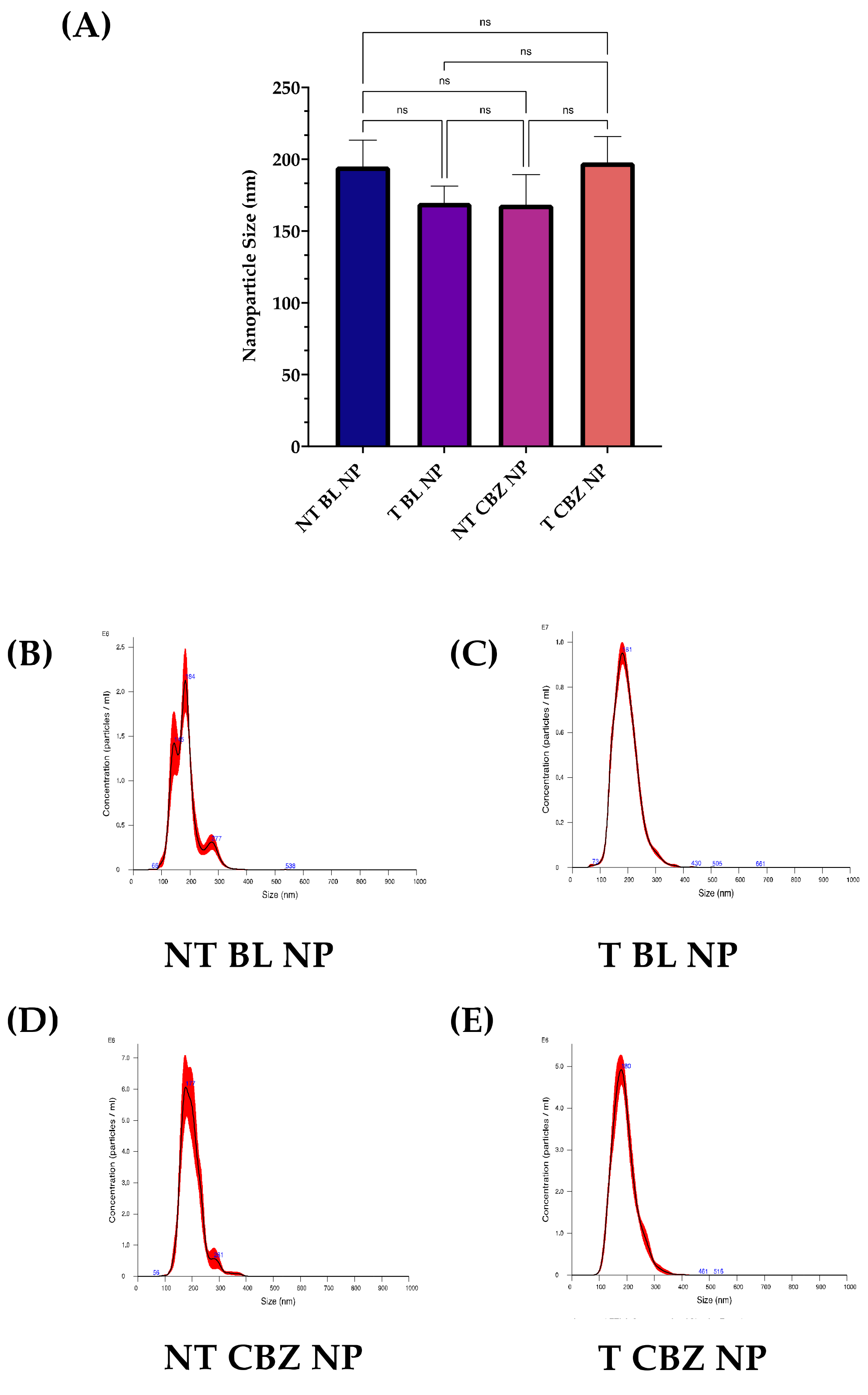

Figure S2. Nanoparticle Size Characterization by Nano Tracking Analysis (NTA). (A) Bar graph showing the average sizes of nanoparticles: NT BL NP , 194.9 nm; T BL NP, 169.7 nm; NT CBZ NP, 168.3 nm; T CBZ NP, 197.9 nm. ((B-E) The representative plots showing size distribution of nanoparticles using NTA. A One-Way ANOVA followed by Tukey's test was used to determine significance. Graphs created with GraphPad Prism. ns= not significant. (n=2)

Table S2. Gene primers for qPCR.

Primer	Sequence
**Akt For**	5'-CTTCGTGAACATTAACGACAGGGCC-3'
**Akt Rev**	5'-AATGGCCACCCTGACTAAGGAGTGG-3'
**Androgen Receptor For**	5'-GGTGAGCAGAGTGCCCTATC-3'
**Androgen Receptor Rev**	5'-GAAGACCTTGCAGCTTCCAC-3'
**Annexin A2 For**	5'-GCCATCAAGACCAAAGGTGT-3'
**Annexin A2 Rev**	5'-TCAGTGCTGATGCAAGTTCC-3'
**E-cadherin For**	5'-TGGCGTCTGTAGGAAGGCA-3'
**E-cadherin Rev**	5'-GGCTCTTTGACCACCGCTCT-3'
**EPCAM For**	5'-GCCAGTGTACTTCAGTTGGTGC-3'
**EPCAM Rev**	5'-CCCTTCAGGTTTTGCTCTTCTCC-3'
**IL-8 For**	5'-GCAGAGGGTTGTGGAGAAGT-3'
**IL-8 Rev**	5'-TGGCATCTTCACTGATTCTTGG-3'
**MIEN1 For**	5'-CAGTGCTGTGAAGGAGCAGT-5'
**MIEN1 Rev**	5'-GACGGCTGTTGGTGATCTTT-3'
**N-cadherin For**	5'-ACCAGGACTATGACTTGAGCC-3'
**N-cadherin Rev**	5'-GGCGTGGATGGGTCTTTCA-3'
**Slug For**	5'-AAGCATTTCAACGCCTCCAAA-3'
**Slug Rev**	5'-GGATCTCTGGTTGTGGTATGACA-3'
**Snail For**	5'-TCGGAAGCCTAACTACAGCGA-3'
**Snail Rev**	5'-AGATGAGCATTGGCAGCGAG-3'
**Twist1 For**	5'-GCCAGGTACATCGACTTCCTCT-3'
**Twist1 Rev**	5'-TCCATCCTCCAGACCGAGAAGG-3'
**Vimentin For**	5'-GCAAAGATTCCACTTTGCGT-3'
**Vimentin Rev**	5'-GAAATTGCAGGAGGAGATGC-3'
**18S For**	5'-CGGTTCCGATGCCCTGAGGCTCTT-3'
**18S Rev**	5'-CCATCCAATCGGTAGTAGCG-3'

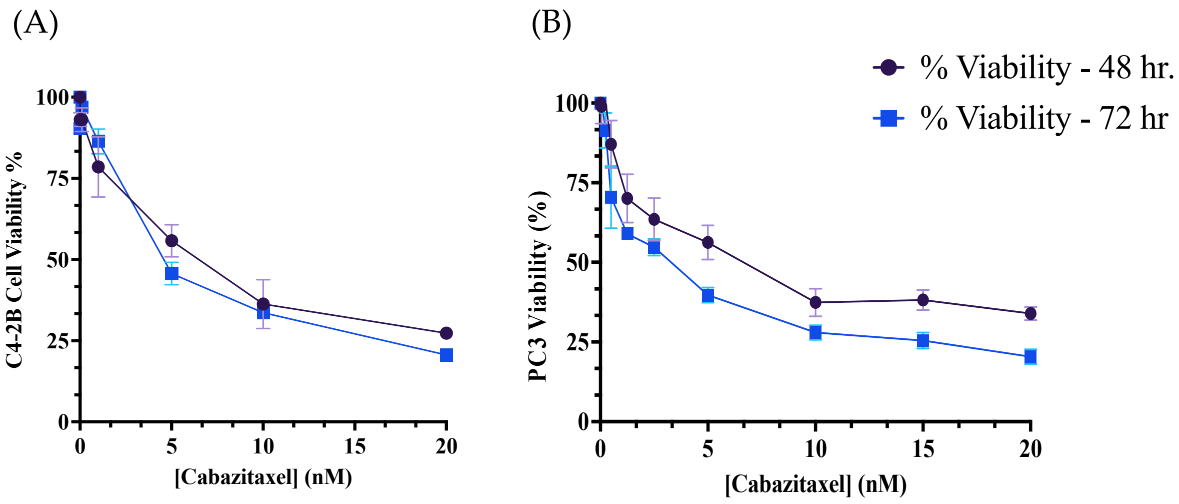

Figure S3. Cabazitaxel Dose Curves for 48 h and 72 h treatments. (A) C4-2B cell line nM concentrations, (B) C4-2B cell line log10 concentrations, (C) PC3 cell line nM concentrations, (D) PC3 cell line log10 concentrations.

Table S3. Summary of IC_{50} concentrations of Cabazitaxel in C4-2B and PC3 prostate cancer cell lines.

Treatment Groups	Cell Line	Cell Line IC_{50} (nM) 48 h	Cell Line IC_{50} (nM) 72 h
CBZ Drug	PC3	3.80	3.45
CBZ Drub	C4-2B	2.80	4.95

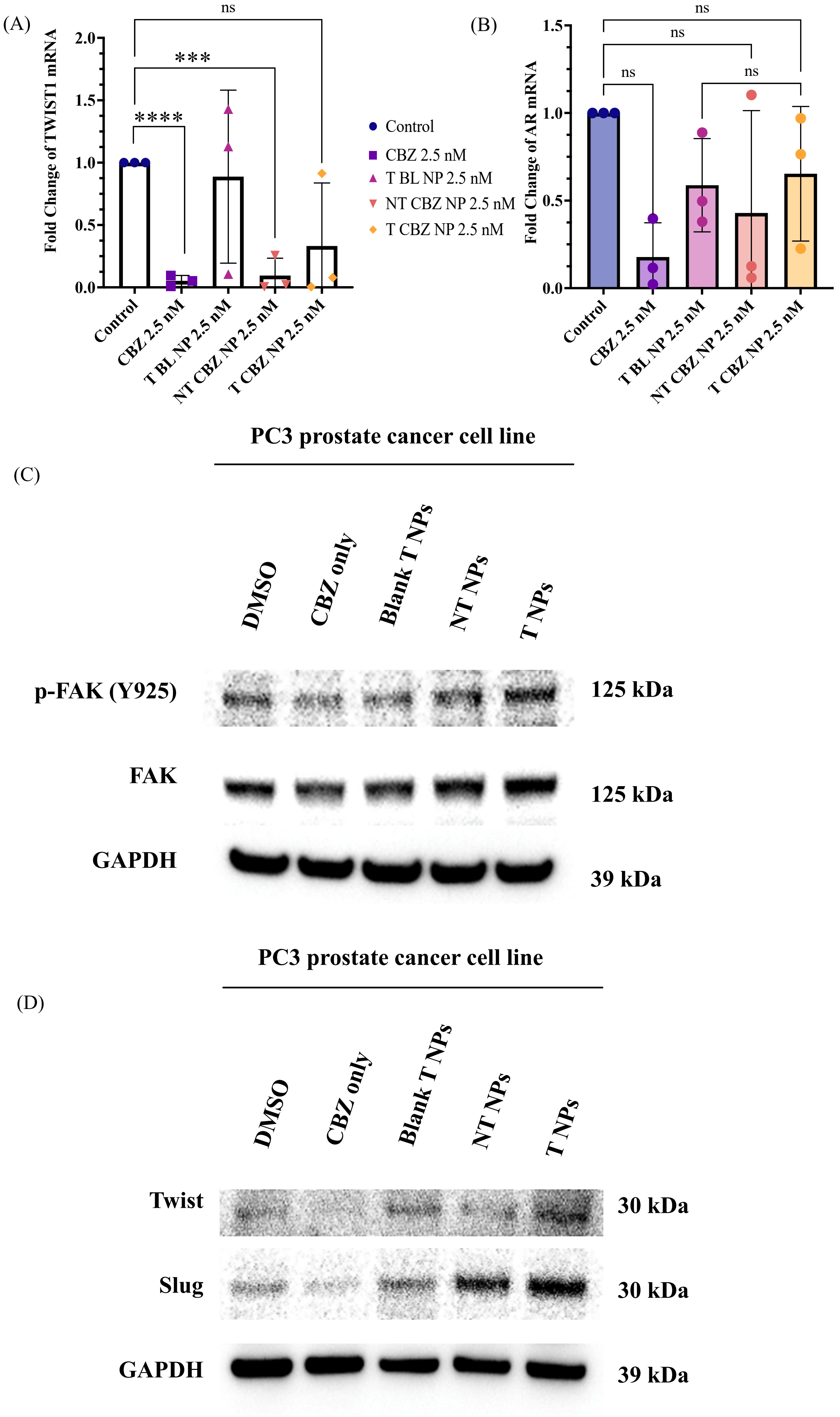

Figure S4. Gene and protein expression in PC3 cell line. (A) Fold change of Twist mRNA. T CBZ NPs did not cause a significant change in Twist gene expression; (B) Fold change of AR mRNA. None of the treatments resulted in a significant inhibition of AR gene expression; (C - D) Protein expression of p-FAK, FAK, Twist, and Slug were not attenuated with CBZ-loaded NP treatments. ns = not significant, ***$p < 0.001$, **** $p < 0.0001$ (n = 3).

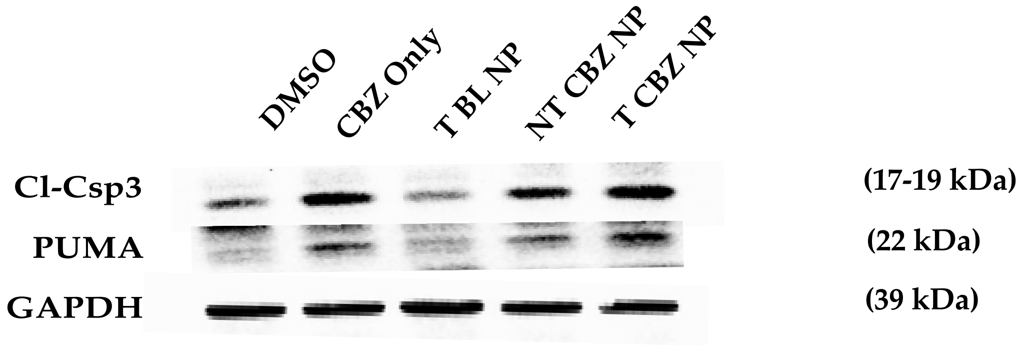

Figure S5. Western blot showing high expression of Cleaved Caspase-3 (Cl-Csp3) and PUMA in PC3 prostate cancer cells treated with cabazitaxel-loaded non-targeted (NT) and targeted (T) nanoparticles.
